# Annotations

**Published:** 1900-08-04

**Authors:** 


### Annotations.

### The British Pharmacopœia.

At the recent meeting of the British Pharmaceutical Conference a good deal was said about the difficulties which have arisen in various quarters from public analysts and others, including magistrates, assuming that the definitions contained in the British Pharmacopœia are to be accepted as the legally recognised standard of purity in regard to all the things mentioned in its pages. The great grievance is in regard to the use of English synonyms, it being maintained by many that the standard fixed by the Pharmacopœia is for "the members of the medical profession" and "those engaged in the preparation of medicines," and that it has nothing to do with the ordinary commercial sale of drugs under their popular names. It is held, for example, that the appropriation by the authors of the Pharmacopœia of the terms "milk of sulphur," "sweet spirit of nitre," and "soft soap" as synonyms for certain pharmacopœial preparations is by no means sufficient to make this work the commercial standard of purity for these articles. The trouble is that the chemists are traders as well as dispensers of medicines. They are competed with on every side, and although they hold that the standard of purity for drugs used in dispensing should be as high as it can be made, they maintain that there are many cases in which drugs which are B.P. articles are used for other than medicinal purposes, and that for such the average of normal or commercial standard of purity meets all the requirements of the case. The president of the Conference said: "To prosecute chemists because, for instance, tincture of myrrh, which is used as a dentifrice rather than as a medicine, or benzoin, which is used in French polish, &c., or soft soap, or ammonium carbonate, soda-water, or other articles in regular household use, do not answer to the tests of purity of the B.P., would constitute an interference with trade that would be as absurd as it would be vexatious." We doubt, however, whether the matter is quite as simple as is implied.

### Public Gardens.

In the hot and parching weather which we have recently passed through, dwellers in London, and especially those who have children, and those who are too old and infirm to stir far away from home, ought many and many a time to have felt thankful to the Metropolitan Public Gardens Association. If any of those happy people who just skim the cream of London's pleasures, who live here only when it is most pleasant to do so, and then when town gets hot, or town gets wet, or town gets otherwise disagreeable, rush to green fields and brighter places, if they will but think what it means to live in London always, and if they will take the map of London issued by the Public Gardens Association and notice what a number of little green oases the association maintains in every part of this great city, we feel sure that they would put their hands in their pockets and give freely to assist in so good a work. Wherever an open space seems to be available, wherever there is an old churchyard or disused burial-ground, a spot where a garden or a recreation-ground could be placed, even a place where a bench could be set up or a tree that might be preserved, there the association steps in, sometimes to do the work itself, sometimes to set in action the local authorities, sometimes to influence private owners, sometimes to stir up districts to a sense of what would be lost to them if some open space which no one thinks about were to be covered with bricks and mortar and lost for ever. Those of us who remembering London thirty or more years ago, recall how few green spots were then to be seen, and noting how comparatively frequent they are now, will recognise what an improvement is thereby effected even from a merely æsthetic point of view, but when we think what a benefit these little gardens, these seats, and these playgrounds are to the poor, the invalids, and the aged, and above all, to the children, we begin to see what a good and useful work is being done by this unpretentious association, and how worthy it is of public support.

### Hospital Politics and the Central Funds.

The success of central funds like the Metropolitan Hospital Sunday Fund has depended largely upon the maintenance from the outset of a policy of non-interference in hospital politics. It is, therefore, matter for regret that this year the Distribution Committee of the Hospital Sunday Fund, which has heretofore deservedly possessed the confidence of the whole hospital world, should have departed from precedent. We believe, from the inquiries we have made, that this abandonment of a sound policy is due to a misapprehension of the facts on the part of a majority of the members of the Distribution Committee. Those facts are set forth in the papers which have been distributed to the Press, from which it appears that the Committee of the National Hospital for the Paralysed have never received any official written intimation from the Distribution Committee with reference to the disagreements between themselves and the medical staff until a few weeks ago. Members of the Distribution Committee appear, from the discussion at the Mansion House, to believe, that a year ago an intimation was sent to the Committee of the National Hospital stating that, although a grant was made in 1899, the Distribution Committee hoped that the disputes in question would be adjusted before the next application for an award from the Hospital Sunday Fund. As matters stand at present, the Council of the Hospital Sunday Fund is committed to a policy of interference in domestic matters concerning the internal economy of the hospitals, although Sir Sydney Waterlow rightly maintained that any such policy was outside the true functions of the Distribution Committee and the Council of the Hospital Sunday Fund. To state this is to show that the resolution passed by the Council of the Sunday Fund on Monday was adopted only because the majority by which it was carried were under a misapprehension as to the true bearings of the question upon which they were recording their votes. Every well-wisher of the hospitals and of the Sunday Fund must desire that an early opportunity may be found to retrace the false step thus taken by the Council by the insertion of a provision in the laws of the Fund which will make it clear that any interference in the politics and domestic concerns of any of the hospitals is *ultra vires* so far as the Council or its Distribution Committee are concerned. We are confident that, could Sir Sydney Waterlow have heard all that passed at the meeting of the Distribution Committee, having regard to his long connection with the Sunday Fund, he would never have supported a policy which, if persisted in, must materially and permanently injure the Hospital Sunday Fund, by depriving it of the neutral position which has so far constituted its chief claim to public confidence.

